# Supplementary material for: Chromothripsis during telomere crisis is independent of NHEJ, and consistent with a replicative origin
Source: Genome Res. 2019 May;29(5):737–49. doi: 10.1101/gr.240705.118 (PMC6499312; doi:10.1101/gr.240705.118)
Supplement: Supplemental Material [file supp_gr.240705.118_Supplemental_file_1.zip › contigs/annotated_contigs/DB46/contig.5.DB46_length_762_mean_cov_7.27296587927.docx]

**DB46_length_762_mean_cov_7.27296587927**

```
TA|CTGAATGAGAAGCCGAGTGACTGAATGAATGTATGAATGAAGGAACAGGTCAGTAACTGGCGGTTCCCGACGTTGGAGCCGACTAA
   >chr15:99791268-99791425 + E=1e-76
ATTACCCCTTCAAGACGCTAAGCAAAAGCCACTCCGTAGACATCAAACCCCACGCCGGAGCCGGAAGCAAG|G|CAAAACACTGAAAAG
                                                                         >chr15:9979677
TTACATTAAGCTTCCCAAGTGCAT|CT|GTGTTCTCGCGCTTTCACGATAGGCGCGGGAAAATGCTCGTTTGCCAAGCGATCTGTGTAT
8-99796819 - E=7e-13     >chr15:99791370-99791537 - E=1e-85
TGGCCGAGAGCGCTACTTCCTCTATGCTGGCCCCGCCCGCGGCTCTACTCTTGCTTCCGGCTCCGGCGTGGGGTTTGATGTCTACGGAG

TGGCTTTTGCTTAGC|CT|TTCTGCATATTAGGCAACTGGAGACC|CAGAG|TGAGACCCTGTCTCTAAAAAAAAATAAGAAGAAAGAA
                  >chr15:99795100-99795131 + E=8e-07 >chr15:99795542-99795910 + E=1e-
AAGAATAAAGAGATAAAGAAAAAAATCAGAATATAGGACAATATTTATATATTAGGAAGTGAAGAAAATGAAGCTATTAGTGCTCTAGA
209
TATAAATAATATATATTTTAATATTTTTGAAGTACCTTAAGCTAAATACAAAATGAGGTAGTTCATAAAAGAAATGGGGTTTGCTGCAT

TTGATTTTTAAGCTTCTCCTTTGGTTGTATGTTATGAAACTGGTCACTGTTTTTCTTACACAAGGTTTCCTATCTCAAGTGCAAAAACT

ATTTTTTTCCATTAGGACTATTTCTAATAAGTAATAAATATTGCTTAATCAGAGTATTT
```
